# Unexpected elevated alanine aminotransferase, asparte aminotransferase levels and hepatitis E virus infection among persons who work with pigs in accra, ghana

**DOI:** 10.1186/1743-422X-7-336

**Published:** 2010-11-22

**Authors:** Andrew A Adjei, Yao Tettey, John T Aviyase, Clement Adu-Gyamfi, Julius A Mingle, Edmund T Nartey

**Affiliations:** 1 Department of Pathology, University of Ghana Medical School, College of Health Sciences, University of Ghana, Accra, Ghana; 2 Department of Microbiology, University of Ghana Medical School, College of Health Sciences, University of Ghana, Accra, Ghana; 3 Kwame Nkrumah University of Science and Technology, Department of Medical Laboratory Sciences, Kumasi, Ghana; 4 Centre for Tropical Clinical Pharmacology and Therapeutics, University of Ghana Medical School, College of Health Sciences, University of Ghana, Accra, Ghana

## Abstract

**Background:**

Several studies have suggested that elevated serum alanine aminotransferase (ALT) and asparte aminotransferase (AST) may be markers of hepatitis E virus (HEV) infection. Thus, individuals with elevated ALT and AST may have ongoing subclinical infection of HEV. We estimated the prevalence of anti-HEV antibodies and serum ALT and AST levels among persons who work with pigs in Accra, Ghana.

**Results:**

Three hundred and fifty- persons who work with pigs provided blood samples for unlinked anonymous testing for the presence of antibodies to HEV, ALT and AST levels. The median age of participants was 32.85 ± 11.38 years (range 15-70 years). HEV seroprevelance was 34.84%. Anti-HEV IgG was detected in 19.26% while anti-HEV IgM was detected in 15.58% of the persons who tested positive. On multivariate analysis, the independent determinants of HEV infection were, being employed on the farm for less than six months [odds ratio (OR) 8.96; 95% confidence interval (95% CI) 5.43-14.80], having piped water in the household and/or on the farm (OR 13.33; 95% CI 5.23-33.93) and consumption of alcohol (OR 4.91: 95% CI 2.65-9.10). Levels >3× the expected maximum were found for both ALT and AST among individuals who tested positive for anti-HEV IgG (ALT, 210.17 ± 11.64 U/L; AST, 127.18 ± 11.12 U/L) and anti-HEV IgM (ALT, 200.97 ± 10.76 U/L; AST, 120.00 ± 15.96 U/L).

**Conclusion:**

Consistent with similar studies worldwide, the results of our studies revealed a high prevalence of HEV infection, ALT and AST values in pig handlers.

## Introduction

Hepatitis E virus (HEV) infection is one of the major cause of human viral disease with clinical and pathological features of acute hepatitis. The infection represents an important public health concern in many developing countries, where it is primarily transmitted through the faecal oral route due to contaminated water and food [1], and is often responsible for epidemic outbreaks [2]. The infection affects primarily young adults and is generally mild, except for women in late pregnancy in whom 20% mortality has been reported [3].

The first animal strain of HEV was characterised in pigs in the United States of America [4,5] and since then several other strains have been described in pigs worldwide [4,6] suggestive that pigs can represent a reservoir of the infection. The identification of a U.S.A. strain of HEV apparently acquired inside the U.S.A. after the isolation of a closely related HEV strain from swine in the same region of the U.S.A. validates that HEV is a zoonotic infection [4,5]. Similar findings have been reported in China [7], South Korea [8] and Japan [9].

Growing evidence suggests that individuals who work in contact with swine such as pig farmers, veterinarians and slaughterhouse workers are at increased risk of acquiring HEV infection [10-12]. We recently reported high prevalence of anti-HEV IgM and IgG among pig handlers in Accra, Ghana [13]. More recently, unpublished reports from the Gastroenterology Unit of the Department of Medicine and Therapeutics, Korle-Bu Teaching Hospital, Accra, Ghana indicate cases of acute hepatitis [with elevated alanine aminotransferase (ALT) and aspartate aminotransferase (AST) levels higher than 200 U/L] without a defined aetiology. Although the physicians did not estimate HEV antibodies in the patients' serum, based on clinical examinations, they speculated that HEV may be one of the causative pathogens.

Several studies [14-17] suggest that elevated serum ALT and AST (> 200 U/L) may be a marker of HEV infection and that individuals with elevated ALT and AST may have ongoing subclinical infection of HEV. HEV infection is likely to be prevalent in Ghana for two reasons. First, various animals that are potential sources of transmission (pigs, sheep, goats, and cattle) share a habitat with humans. Second, the common sources of drinking water, including tap water, may be contaminated because of the inadequacy of standard water treatment measures to remove the organism. Here we report the results of a 10-month study of the prevalence of anti-HEV antibodies and serum ALT and AST levels among persons who work with pigs. In this study, we also examined the association of HEV with various suggested risk factors for its transmission.

## Materials and methods

### Study Site

A cross-sectional study was carried out between the months of January and October 2008 among workers in 6 commercial pig farms in the Greater Accra Region of Ghana. The pig rearing facilities used for the study ranged from small family-run piggeries (~200 pigs) to large-scale pig farming operations (~4000 pigs) where animal housing conditions, sanitation and overall management were generally of a lower standard. All the farms are situated within the communities in high population density areas. Two of the farms are close to each other while the rest of the farms are about 95 km from the other two farms. The study was approved by the Ethical and Protocol Review Committee of the University of Ghana Medical School, Accra, Ghana.

### Study Population

Subjects for the study were male workers of the Farms. The study population was of similar socio-economic and cultural backgrounds. In general, participants had been residing in their respective communities for most part of their lives. Farming is the major source of income; most farmers rear pigs, and other domestic animals such as goats, sheep, cows and poultry for their own consumption and for sale to supplement the family incomes. After an explanation of the purpose of the study, all the workers were invited to participate. They were informed that the study was confidential and that information provided by them would not affect their employment status. A total of 353 persons joined in the research. Written and informed consent was obtained from each participant, and the information regarding the protocol and informed consent was presented at the appropriate literacy level. The study was conducted in a confidential manner and random unique study generated numbers were employed to identify the participants.

### Questionnaire

All the 353 consenting participants completed a structured questionnaire assessing socio-demographic characteristics, and risk factor profile for the infection under investigation.

### Sample Collection and Serological Tests

Blood (10 ml) samples were collected from each of the consenting participants in plain tubes. Samples were centrifuged and the sera kept frozen at -20°C until analysed. Sera were tested at the Public Health Reference Laboratories, Korle-Bu, Accra, Ghana, for the presence of IgM and IgG antibodies (using ELISA Kit produced by International Immuno-Diagnostics, CA, U.S.A.) to HEV, in accordance with the manufacturer's instructions. The results were scored as positive or negative according to the standard procedures recommended by the manufacturer. Positive and negative controls were included in all the ELISA microplates assayed. The International Immuno-Diagnostics assays were chosen for this investigation because our validation exercises (data not shown) had demonstrated these assays to be sensitive and specific than some other commercially available assays. Liver function tests, particularly ALT (normal range, 13-69 IU/L) and AST (normal range, 13-46 IU/L) in serum were determined at the Chemical Pathology Department, Korle Bu Teaching Hospital Central Laboratory, Accra, Ghana, using the Thermo Spectronic spectrophotometer (Helios, Barcelona, Spain).

### Statistical analysis

The Statistical Analysis Software (SAS Institute, Cary, NC, USA) version 9.1 was used to complete all data analyses. For each generally accepted risk factor for HEV infection, the odds ratio (OR) and the 95% confidence interval (95% CI) were calculated to assess associations with sociodemographic and behavioural variables in univariate analysis. A P value of < 0.05 was considered significant. Independent determinants of anti-HEV reactivity were evaluated by calculating the adjusted OR by univariate and multivariate analysis for the risk factor variables found to be significant and to estimate their joint influence.

## Results

### Study population

To examine whether persons who work with pigs are at high risk for HEV infection, 353 subjects (mean age 32.85 ± 11.38 years, median age 33 years, modal age 28 years, age range 15 to 70 years), who had been handling pigs for more than 3 months in 6 pig farms in Accra, were recruited to participate in the study between January and October, 2008. The serum samples were assayed for the presence of anti-HEV IgG, anti-HEV IgM, ALT and AST. All the subjects were occupationally exposed to the pigs (feeding the pigs, cleaning barns, assisting the sows at birth and butchering on the farm). Of the subjects occupationally exposed to the pigs, 39.94% (141 out of 353) had been working in the same farm setting for less than 6 months whilst 60.06% (212 out of 353) had been working in the same farm setting for more than 6 months.

### Prevalence of serum anti-HEV IgM in pig handlers

The overall prevalence of anti-HEV was 34.84% (123 out of 353). Of the persons who tested positive, anti-HEV IgG was detected in 19.26% (68 out of 123) while anti-IgM was detected in 15.58% (55 out of 123). There was a significant difference between them, P < 0.05.

Table 1 shows HEV sero-positivity and age distribution among the pig handlers. There was no correlation between increasing age and either HEV-IgG or HEV-IgM seropositivity among persons who work with pigs.

**Table 1:** Age-specific Prevalence of Anti-HEV IgG and Anti-HEV IgM Among Pig Handlers.

		IgG			IgM		
Age (yrs)	N	Positive	Negative	p-value	Positive	Negative	p-value
					
		n (%)	n (%)		n (%)	n (%)	
≤20	41	6 (14.63)	35 (85.37)	0.001	7 (17.07)	34 (82.93)	< 0.001
21-25	55	12 (21.82)	43 (78.18)	< 0.001	7 (12.73)	48 (87.27)	< 0.001
26-30	82	16 (19.51)	66 (80.49)	< 0.001	15 (18.29)	67 (81.71)	< 0.001
31-35	56	12 (21.43)	44 (78.57)	< 0.001	5 (8.93)	51 (91.07)	< 0.001
36-40	38	4 (10.53)	34 (89.47)	< 0.001	9 (23.68)	29 (76.32)	0.002
41-45	37	8 (21.62)	29 (78.38)	0.001	6 (16.22)	31 (83.78)	< 0.001
46-50	13	3 (23.08)	10 (76.92)	0.092	1 (7.69)	12 (92.31)	0.003
51-55	14	5 (35.71)	9 (64.29)	0.429	0 (0)	14 (100)	< 0.001*
56-60	6	0 (0)	6 (100)	0.031*	2 (33.33)	4 (66.67)	0.688
> 60	11	2 (18.18)	9 (81.82)	0.065	3 (27.27)	8 (72.73)	0.277

*Binomial test used instead of Chi-square test; N values indicate number of responses obtained in each category

However, the overall prevalence of antibodies to HEV-IgG was highest (35.71%) among persons 51-55 years of age, followed by 23.08% in 46-50 year group, then 21.82% in 21-25 year group (Table 1). Similarly, the overall prevalence of antibodies to HEV-IgM was highest (33.33%) among persons 56-60 years of age, followed by 23.68% in 36-40 year group, then 27.27% in > 60 year group (Table 1). Tables 2 and 3 show test results for ALT and AST among persons who work with pigs in the different age groups.

**Table 2:** Comparison of Age-specific Distribution of HEV Sero-reactivity (IgG) and ALT Levels Among Pig Handlers.

		Number of ALT values				
					
Age (yrs)	IgG status	> 69 U/L	≤69 U/L	OR	95% CI	P-value
≤20 (n = 41)	*Positive*	2	4	Not estimable	Not estimable	0.018
	*Negative*	0	35	1.00	Baseline	

21-25 (n = 55)	*Positive*	4	8	21.00	2.07-213.28	0.006
	*Negative*	1	42	1.00		

26-30 (n = 82)	*Positive*	8	8	65.00	7.17-589.42	0.001
	*Negative*	1	65	1.00	Baseline	

31-35 (n = 56)	*Positive*	3	9	7.00	1.02-48.16	0.060
	*Negative*	2	42	1.00	Baseline	

36-40 (n = 38)	*Positive*	1	3	5.33	0.37-77.50	0.291
	*Negative*	2	32	1.00	Baseline	

41-45 (n = 37)	*Positive*	3	5	8.10	1.07-61.54	0.057
	*Negative*	2	27	1.00	Baseline	

46-50 (n = 13)	*Positive*	1	2	2.00	0.12-34.82	1.000
	*Negative*	2	8	1.00	Baseline	

51-55 (n = 14)	*Positive*	1	4	Not estimable	Not estimable	0.357
	*Negative*	0	9	1.00	Baseline	

56-60 (n = 6)	*Positive*	0	0	Not estimable	Not estimable	Not estimable
	*Negative*	0	6	1.00	Baseline	

> 60 (n = 11)	*Positive*	0	2	Not estimable	Not estimable	Not estimable
	*Negative*	0	9	1.00	Baseline	

OR, odds ratio; CI, confidence interval; n values indicate number of responses obtained in each category

**Table 3:** Comparison of Age-specific Distribution of HEV Sero-reactivity (IgM) and ALT levels among pig handlers.

		Number of ALT values				
					
Age (yrs)	IgM status	> 69 U/L	≤69 U/L	OR	95% CI	p-value
≤ 20 (n = 41)	*Positive*	4	3	44.00	3.65-530.55	0.002
	*Negative*	1	33	1.00	Baseline	

21-25 (n = 55)	*Positive*	5	2	37.50	5.01-280.90	< 0.001
	*Negative*	3	45	1.00	Baseline	

26-30 (n = 82)	*Positive*	9	6	99.00	10.66-919.41	< 0.001
	*Negative*	1	66	1.00	Baseline	

31-35 (n = 56)	*Positive*	3	2	36.75	3.76-359.45	0.003
	*Negative*	2	49	1.00	Baseline	

36-40 (n = 38)	*Positive*	5	4	Not estimable	Not estimable	< 0.001
	*Negative*	0	29	1.00	Baseline	

41-45 (n = 37)	*Positive*	3	3	Not estimable	Not estimable	0.003
	*Negative*	0	31	1.00	Baseline	

46-50 (n = 13)	*Positive*	1	0	Not estimable	Not estimable	0.077
	*Negative*	0	12	1.00	Baseline	

51-55 (n = 14)	*Positive*	0	0	Not estimable	Not estimable	Not estimable
	*Negative*	0	14	1.00	Baseline	

56-60 (n = 6)	*Positive*	2	0	Not estimable	Not estimable	0.467
	*Negative*	2	2	1.00	Baseline	

> 60 (n = 11)	*Positive*	3	0	Not estimable	Not estimable	0.182
	*Negative*	3	5	1.00	Baseline	

OR, odds ratio; CI, confidence interval; n values indicate number of responses obtained in each category

Our analysis show that levels >3× the expected maximum were found for both ALT and AST among individuals who tested positive for anti-HEV IgG (ALT, 210.17 ± 11.64 IU/L vs. anti-HEV negative, ALT, 120.30 ± 50.55 IU/L: AST, 127.18 ± 11.12 vs. anti-HEV negative, 60.921 ± 16.04, P < 0.001) and anti-HEV IgM (ALT, 200.97 ± 10.76 IU/L vs. anti-HEV negative, 93.33 ± 19.17 IU/L vs. AST, 120.00 ± 15.96; anti-HEV negative, 60.92 ± 16.04, P < 0.001). Compared to the different age groups, a higher number of pig handlers, 26-30 years of age, who tested positive for either anti-HEV IgM or anti-HEV IgG had ALT (anti-IgG, 8 out of 23, Table 2; anti-IgM, 9 out of 35, Table 3) and AST (anti-IgG, 6 out of 17, Table 4; anti-IgM, 4 out of 14, Table 5) levels above the expected maximum.

**Table 4:** Comparison of Age-specific Distribution of HEV Sero-reactivity (IgG) and AST Levels Among Pig Handlers.

		Number of AST values				
					
Age (yrs)	IgG status	> 46 U/L	≤46 U/L	OR	95% CI	p-value
≤20 (n = 41)	*Positive*	1	5	Not estimable	Not estimable	0.146
	*Negative*	0	35	1.00	Baseline	

21-25 (n = 55)	*Positive*	3	9	Not estimable	Not estimable	0.008
	*Negative*	0	43	1.00	Baseline	

26-30 (n = 82)	*Positive*	6	10	19.20	3.39-108.68	0.001
	*Negative*	2	64	1.00	Baseline	

31-35 (n = 56)	*Positive*	3	9	Not estimable	Not estimable	0.008
	*Negative*	0	44	1.00	Baseline	

36-40 (n = 38)	*Positive*	0	4	Not estimable	Not estimable	1.000
	*Negative*	2	32	1.00	Baseline	

41-45 (n = 37)	*Positive*	3	5	3.75	0.63-22.20	0.308
	*Negative*	4	25	1.00	Baseline	

46-50 (n = 13)	*Positive*	1	2	2.00	0.12-34.82	1.000
	*Negative*	2	8	1.00	Baseline	

51-55 (n = 14)	*Positive*	0	5	Not estimable	Not estimable	Not estimable
	*Negative*	0	9	1.00	Baseline	

56-60 (n = 6)	*Positive*	0	0	Not estimable	Not estimable	Not estimable
	*Negative*	0	6	1.00	Baseline	

> 60 (n = 11)	*Positive*	0	2	Not estimable	Not estimable	0.564
	*Negative*	3	6	1.00	Baseline	

OR, odds ratio; CI, confidence interval; n values indicate number of responses obtained in each category

**Table 5:** Comparison of Age-specific Distribution of HEV Sero-reactivity (IgM) and AST Levels Among Pig Handlers.

		Number of AST values				
					
Age (yrs)	IgM status	> 46 U/L	≤46 U/L	OR	95% CI	p-value
≤20 (n = 41)	*Positive*	2	5	Not estimable	Not estimable	0.026
	*Negative*	0	34	1.00	Baseline	

21-25 (n = 55)	*Positive*	3	4	4.39	0.80-24.00	0.104
	*Negative*	7	41	1.00	Baseline	

26-30 (n = 82)	*Positive*	4	11	3.12	0.78-12.47	0.202
	*Negative*	7	60	1.00	Baseline	

31-35 (n = 56)	*Positive*	0	5	Not estimable	Not estimable	1.000
	*Negative*	2	49	1.00	Baseline	

36-40 (n = 38)	*Positive*	0	9	Not estimable	Not estimable	Not estimable
	*Negative*	0	29	1.00	Baseline	

41-45 (n = 37)	*Positive*	0	6	Not estimable	Not estimable	Not estimable
	*Negative*	0	31	1.00	Baseline	

46-50 (n = 13)	*Positive*	1	0	Not estimable	Not estimable	0.231
	*Negative*	2	10	1.00	Baseline	

51-55 (n = 14)	*Positive*	0	0	Not estimable	Not estimable	Not estimable
	*Negative*	3	11	1.00	Baseline	

56-60 (n = 6)	*Positive*	2	0	Not estimable	Not estimable	Not estimable
	*Negative*	4	0	1.00	Baseline	

> 60 (n = 11)	*Positive*	2	1	Not estimable	Not estimable	0.055
	*Negative*	0	8	1.00	Baseline	

OR, odds ratio; CI, confidence interval; n values indicate number of responses obtained in each category

Persons 26-30 years of age who work with pigs and who tested positive to antibodies to HEV IgG had a 14.06 fold (95% CI 6.27-31.49) and a 6.97 fold (95% CI 3.19-15.24) higher risk of elevated ALT and AST levels, respectively. Similarly, pig handlers 26-30 years of age who tested positive to antibodies to HEV IgM had a 41.71 fold (95% CI 18.80-92.56) and a 3.73 fold (95% CI 1.79-7.75) higher risk of increased ALT and AST levels, respectively.

### Association of risk factors with anti HEV infection

Potential risk factors for infection were examined, to determine whether there were associations with anti-HEV prevalence.

As shown in Table 6, anti-HEV prevalence was significantly higher (P < 0.001) among persons who work with pigs and had been working in the same farm setting for less than 6 months compared to persons who work with pigs and had been working in the same farm setting for more than 6 months (%, 89 out of 123 vs, %, 34 out 123 respectively). Pig handlers who had been working in the same farm setting for less than 6 months had a 8.96 fold higher risk of HEV infection as compared with those who had been working in the same farm setting for more than 6 months (95% CI 5.43-14.80).

**Table 6:** Odds ratio for HEV sero-reactivity and corresponding 95% CI according to risk factors

Characteristic	HEV positive	HEV negative	OR	95% CI	p-value
**Piped-water (n = 353)**					
Yes	118	147	13.33	5.23-33.93	< 0.001
No	5	83	1.00	Baseline	

**Cleaning barns (n = 353)**					
Yes	101	111	4.92	2.90-8.35	< 0.001
No	22	119	1.00	Baseline	

**Assisting sows at birth (n = 353)**					
Yes	98	129	3.07	1.84-5.11	< 0.001
No	25	101	1.00	Baseline	

**Butchering at farm (n = 353)**					
Yes	89	109	2.91	1.81-4.66	< 0.001
No	34	121	1.00	Baseline	

**Eating under-cooked pork (n = 353)**					
Yes	22	19	2.42	1.25-4.67	0.009
No	101	211	1.00	Baseline	

**Reported cut with blood- blood contact (n = 353)**					
Yes	95	184	0.85	0.50-1.44	0.584
No	28	46	1.00	Baseline	

**Level of formal education (n = 353)**					
Tertiary	0	3	Not estimable	Not estimable	0.554
Senior high	2	8	0.56	0.11-2.70	0.518
Basic	62	88	1.56	1.00-2.45	0.053
None	59	131	1.00	Baseline	

**Length of time on farm (n = 353)**					
≤6 months	89	52	8.96	5.43-14.80	< 0.001
> 6 months	34	178	1.00	Baseline	

OR, odds ratio; CI, confidence interval; n values indicate number of responses obtained in each category

A similar pattern was observed among persons who work with pigs and had water piped into their homes and/or on the farms and clean barns, assist sows at birth, butcher pigs at the farm, and eat pork (undercooked pork). As shown in Table 6, such persons had higher prevalence of HEV infection. Among the pig handlers, the greatest risk of HEV seropositivity was strongly associated with those who had water piped into their homes and/or on the farms (OR 13.33; CI 5.23-33.93), and, to a lesser extent, those who had close contact with pigs, such as cleaning barns (OR 4.92; CI 2.90-8.35), assisting sows at birth (OR 3.07; CI 1.84-5.11), and butchering pigs at the farm (OR 2.91; CI 1.81-4.66), and eat under-cooked pork (OR 2.42; CI 1.25-4.67). Interestingly, there was no association (OR 0.85; CI 0.50-1.44) of anti-HEV reactivity with persons who work with pigs who had reported having a history of a cut with blood-to-blood contact (Table 6). Similarly, there was no association (OR 0.56; CI 0.11-2.70) of anti-HEV reactivity with persons who work with pigs and level of education.

Of the pig handlers who tested positive to antibodies to HEV, alcohol was consumed by 88.61% (109 out of 123). Our analysis indicated association with alcohol consumption. In univariate analysis, persons who work with pigs and consume alcohol, were at an increased risk (OR 4.91: 95% CI 2.65-9.10) of HEV infection. Pig handlers who consume alcohol above the recommended weekly intake (defined as a maximum weekly intake of 21 units) had a 3.48 fold (95% CI 1.96-6.17) higher risk of HEV infection. In a multivariate analysis, HEV infection was independently associated with length of time a person has been working on the farm, having piped water into their homes and/or the farms and consumption of alcohol.

## Discussion

This is the first study in Ghana reporting ALT, AST values and risk factors associated with HEV infection in persons who work with pigs, and demonstrates the high prevalence of and the considerable potential for the transmission of HEV infection in pig farms in Ghana. Although there is no report from the Ministry of Health, Accra, Ghana indicating that Ghana is an endemic area for hepatitis E, this study found very high overall prevalence rates (34.84%) of HEV antibody. In addition, the study found significantly high ALT and AST levels >3× the expected maximum among persons occupationally exposed to pigs, suggesting the possibility of subclinical infections in the country. Further studies with a larger number of pig handlers will be necessary to draw a definitive conclusion. Moreover, additional studies need to be done to isolate both the pig and human HEV strains in Ghana and determine the genomic relationship between them if both the human and pig HEV are isolated.

The risk of HEV infection correlated with length of time employed, close contact with animals or animal waste, such as cleaning barns or assisting sows at birth, butchering pigs, consumption of alcohol, and having piped water in homes and/or on the farms. The finding of higher HEV antibody prevalence in persons who work with pigs in Ghana is consistent with literature, and is widely attributable to work-related behaviours practised on the farm settings, although transmission of HEV has also been documented among individuals outside the farm setting and persons who are not occupationally exposed to pigs [18-22]. The overall seroprevalence of HEV infection among persons who work with pigs in Ghana (34.84%) is higher than the results of similar studies in persons who work with pigs in Taiwan [10] (26.7%) but comparable to the reported seroprevalence of 51.1% in persons who work with pigs in Moldova [11]. The increased seroprevalence of HEV in persons who work with pigs (34.84% %) in Ghana suggests that HEV may be widespread in pig populations in the country and therefore reasonable to speculate that HEV may circulate in the general population. In addition, because the virus is transmitted through the faecal-oral route, transmission of HEV is greatly dependent on the sanitary conditions under which the pig handlers work. In Ghana, there are great social differences and sanitary conditions are quite precarious in many areas. The sanitary conditions at the work place during the period of study were very deplorable and all the farms were situated in densely populated areas where the animals share their habitat with humans. Of interest, persons who work with pigs that had water piped into their homes and/or on the farms had higher anti-HEV reactivity than those who do not have piped water into their homes and/or on the farms (33.43%; 118 out of 353 vs. 0.14%; 5 out of 353%, respectively; Table 6). This finding is not surprising since most of the water delivery pipes are broken and/or exposed and as such the water may have been easily contaminated with faecal effluent in the locality. Moreover, hand-washing facilities are not easily available on the farms.

Another finding of interest reported herein in our study is that anti-HEV prevalence was significantly higher (P < 0.001) among persons who had been working with pigs in the same farm setting for less than 6 months compared to those who had been working in the same farm setting for more than 6 months (63.12%, 89 out of 141 vs, 16.04%, 34 out 212 respectively). The reason(s) for this disparity cannot be discerned from our study. However, it was observed that newly recruited individuals spend more time cleaning barns and assisting sows at birth, and this perhaps may have accounted for the high seroprevalence rate of HEV infection. Further studies need to be done to define the high prevalence of anti-HEV antibodies in such population.

Of particular note, pig handlers studied who tested positive for antibodies to IgG anti-HEV or IgM anti-HEV had ALT (210.17 ± 11.64 IU/L, 200.97 ± 10.76, IU/L, respectively) levels >3× the expected maximum. A similar pattern was noted in serum AST levels among pig handlers. Levels 3× the expected maximum were found in pig handlers studied who tested positive for antibodies to IgG anti-HEV (AST, 127.18 ± 11.12) or IgM anti-HEV (AST, 120.00 ± 15.96). Interestingly, among the different age groups, pig handlers, 26-30 years of age, had higher levels of ALT and AST (data not shown).

Growing evidence suggest that elevated ALT and AST levels are associated with recent acute HEV infection [14-17,23]. Similar results were obtained in our study and thus provided a unique opportunity to diagnose asymptomatic and symptomatic HEV infection in an occupationally exposed group. The presence of seropositive IgM/IgG anti-HEV and increased levels of ALT and AST usually indicate recent HEV infection [23] and may signify recent introduction of HEV into these farms. There is therefore the need to investigate other farms around these locations to ascertain whether there had been any earlier infections among farm workers. There is also the need for further studies to define the clinical and epidemiological importance and pathogenesis of HEV infection in this population.

Another finding of interest reported herein in our study is that anti-HEV prevalence was associated with consumption of alcohol (OR 4.91: 95% CI 2.65-9.10) among persons who had been working with pigs. Although excess alcohol consumption could compromise hepatic function and predispose pig handlers to HEV infection as suggested by our study, consumption of alcohol may not be probably linked to exposure to HEV infection. However, from our studies it is also possible that excess consumption of alcohol may have resulted in lack of self control therefore leading to higher risk behaviour of HEV infection. Further studies need to be conducted to define the link between alcohol consumption and HEV infection among persons who work with pigs.

The small sample size and our inability to test for anti-HEV reactivity in pigs, may be the limitations of this study. However, the detection and prevalence of HEV infection coupled with significantly high values of ALT and AST in persons who work with pigs in Ghana may reflect the prevalence of past and recent HEV infections among pig handlers in the country. Further studies need to be done to define clearly the natural history of HEV infection and transmission in Ghana in order to effectively control and prevent HEV zoonosis.

The results reported herein have significant implications for veterinarians, public health officials, persons who work with pigs and farm managers, and suggest urgent need for the introduction of policies to prevent the transmission of HEV on the farms and the general population. These policy strategies must include increasing education of persons who work with pigs about the need for HEV testing and prevention in infected pig handlers. The implementation of a HEV infection prevention programme in pig farms in Ghana should be seen as an opportunity to improve the health status of the infected persons who work with pigs and to prevent further transmission of HEV, within and without the farm settings. The argument for HEV testing among persons who work with pigs in Ghana is compelling, because of the precarious sanitary conditions in most urban and rural areas, increased incidence of acute viral hepatitis without a defined aetiology (unpublished data, Department of Medicine and Therapeutics, KBTH), and the high infant and maternal mortality. Our findings re-emphasize the suggestion that targeting high-risk pig handlers or universal testing in high prevalence areas, which includes Ghana, could identify most pig handlers, pregnant women, and blood donors infected with HEV at a relatively low cost [1,23].

## Conclusion

This is the first study in Ghana reporting a high prevalence of IgG, IgM anti-HEV antibodies and elevated ALT and AST levels in persons who are occupationally exposed to pigs. The high prevalence of HEV infection coupled with the elevated ALT and AST values suggest that HEV infection should be treated as an occupational illness in persons who work with pigs in Ghana and therefore suggest the urgent need for the introduction of some of the range of effective preventive strategies employed in pig farm settings elsewhere.

## Consent

Fully informed consent was obtained from each study subject. When study subjects were younger than 18 years, informed consent was obtained from their parents.

## Competing interests

The authors declare that they have no competing interests.

## Authors' contributions

AAA, YA, JTA, CAG, JAA, ETN conceived of the study, participated in the design and coordination. All the authors read and approved the final manuscript.
